# Targeting NLRP3 Inflammasome in Translational Treatment of Nervous System Diseases: An Update

**DOI:** 10.3389/fphar.2021.707696

**Published:** 2021-08-30

**Authors:** Qingying Yu, Tingting Zhao, Molin Liu, Duo Cao, Jiaxin Li, Yanling Li, Mengyao Xia, Xiaoyu Wang, Tingting Zheng, Chuanguo Liu, Xiangyu Mu, Peng Sun

**Affiliations:** ^1^ School of Pharmacy, Shandong University of Traditional Chinese Medicine, Jinan, China; ^2^ School of Foreign Languages, Shandong University of Traditional Chinese Medicine, Jinan, China; ^3^ College of Life Science, Yan’an University, Yan’an, China; ^4^ Innovation Research Institute of Chinese Medicine, Shandong University of Traditional Chinese Medicine, Jinan, China; ^5^ School of Traditional Chinese Medicine, Shandong University of Traditional Chinese Medicine, Jinan, China

**Keywords:** NLRP3, inflammasome, nervous system disease, depression, Alzheimer’s disease, ischemic stroke, intracranial haemorrhage

## Abstract

Neuroinflammatory response is the immune response mechanism of the innate immune system of the central nervous system. Both primary and secondary injury can activate neuroinflammatory response. Among them, the nucleotide-binding oligomerization domain-like receptor protein 3 (NLRP3) inflammasome plays a key role in the inflammatory response of the central system. Inflammasome is a type of pattern recognition receptor, a cytoplasmic polyprotein complex composed of members of the Nod-like receptor (NLR) family and members of the pyrin and HIN domain (PYHIN) family, which can be affected by a variety of pathogen-related molecular patterns or damage-related molecular patterns are activated. As one of the research hotspots in the field of medical research in recent years, there are increasing researches on immune function abnormalities in the onset of neurological diseases such as depression, AD, ischemic brain injury and cerebral infarction, the NLRP3 inflammasome causes the activated caspase-1 to cleave pre-interleukin-1β and pre-interleukin-18 into mature interleukin-1β and interleukin-18, in turn, a large number of inflammatory factors are produced, which participate in the occurrence and development of the above-mentioned diseases. Targeted inhibition of the activation of inflammasomes can reduce the inflammatory response, promote the survival of nerve cells, and achieve neuroprotective effects. This article reviews NLRP3 inflammasome’s role in neurological diseases and related regulatory mechanisms, which providing references for future research in this field.

## Introduction

A growing body of evidence has shown that inflammation plays a crucial role in the occurrence and development of various nervous system diseases. So far, the inflammasomes discovered in the study are mainly composed of some members of the NOD-like receptor (NLR) family (NLRP1, NLRC4, NLRP, NAIP5, NLRC5) or inflammatory cysteine-requiring aspartate protease-1 (caspase-1) and cytoplasmic DNA sensor absent in melanoma 2 (AIM-2). Some inflammasome structures also have a poptosis-associated speck-like protein containing a card (ASC).

**FIGURE 1:**
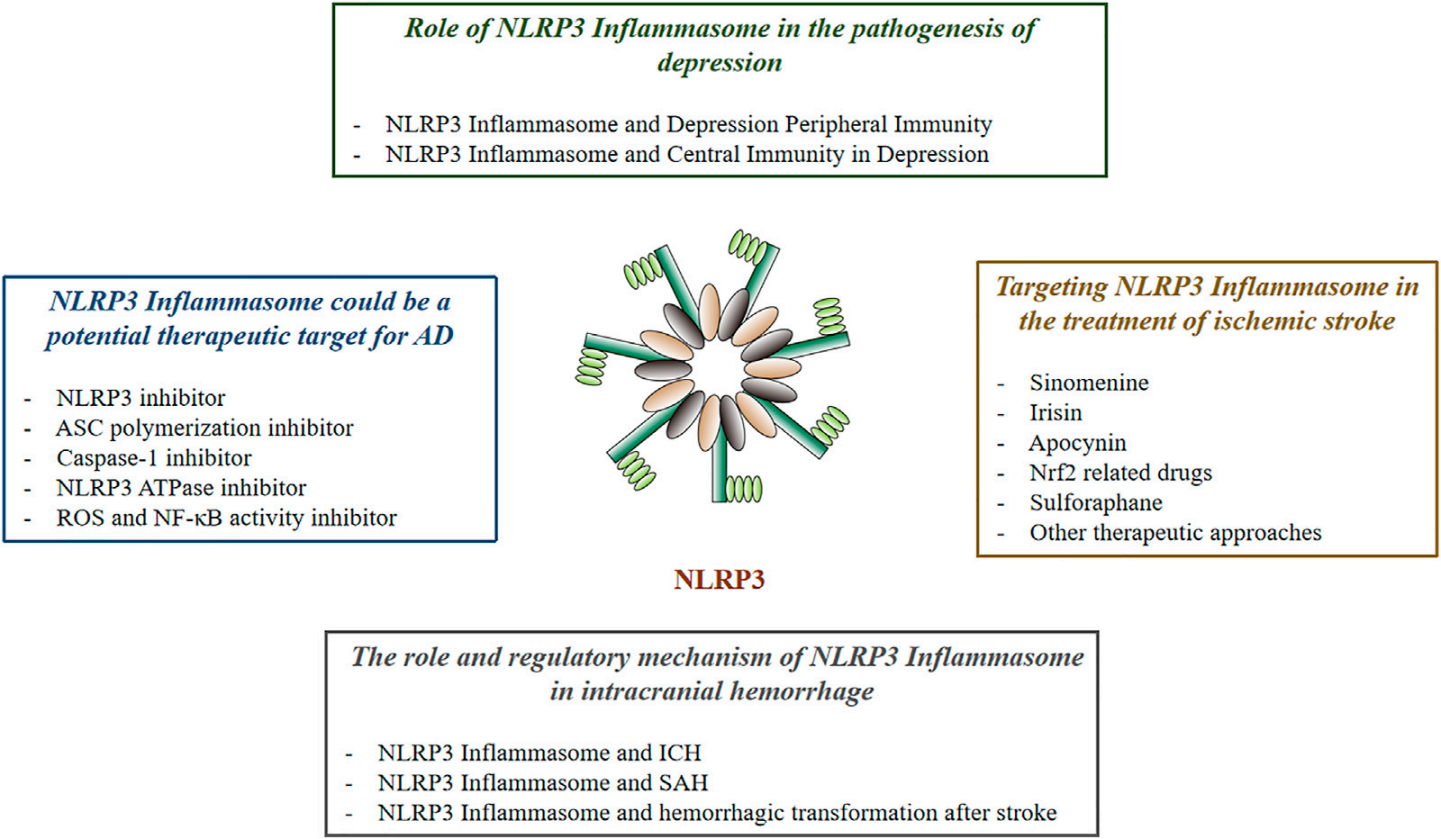
The Effect of NLRP3 in Neurological Diseases.

As a core part of the inflammatory response, inflammasome has attracted much attention. Pathogens of PAMP release of DAMP and physical injury can activate various inflammasome. One of NLRP3 inflammasome has been shown to be involved in the development of a variety of inflammatory-related diseases, such as type 2 diabetes (Vandanmagsar et al., 2011), gout (Dalbeth et al., 2021), atherosclerosis (Karasawa and Takahashi, 2017), neurodegenerative diseases (Freeman and Ting, 2016), cancer (Zhiyu et al., 2016), and inflammatory bowel disease (Opipari and Franchi, 2015). This article reviews the relationship between NLRP3 inflammasome and nervous system diseases (Figure 1).

## NLRP3 Signaling

**FIGURE 2:**
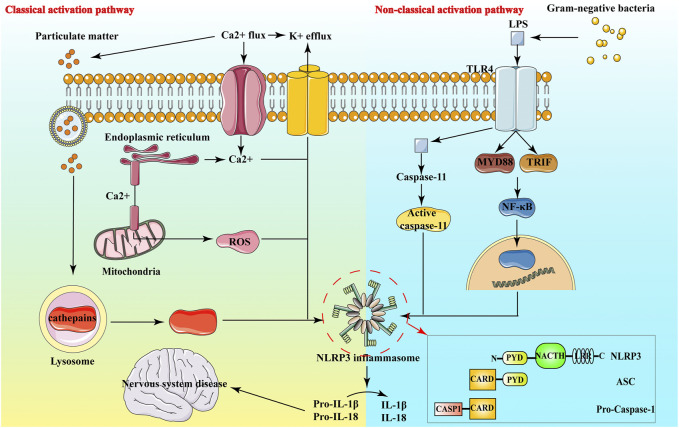
Structure and Activation pathway of NLRP3.

In neurological diseases, it has been found that NLRP3 inflammasome is involved in the neuroinflammatory response of Alzheimer’s Disease (AD) (Heneka et al., 2013), ischemic stroke (FANN et al., 2013), multiple sclerosis (Gris et al., 2010) and other diseases, and is closely linked to the development of these diseases (Choi and Ryter, 2014). The NLRP3 inflammasome expresses in macrophages, neuronal cells, astrocytes and microglia, and exists in the cytoplasm of these cells (Fan et al., 2017). The NLRP3 inflammasome comprises NLRP3 protein, adaptor apoptosis-related spotty protein and effector protein caspase-1 precursor protein (Figure 2). Through their interactions, these three proteins closely regulate the function of inflammasomes (Abderrazak et al., 2015).

NLRP3 belongs to the NLRs protein family, which contains 22 human proteins and at least 34 murine proteins. The structure of most NLRs consists of three parts: the N-terminal Caspase recruitment region (CARD), pyrin domain (PYD), acidic transactivation domain or baculovirus inhibitor of apoptosis protein repeat domain (BIR), mainly mediate the interaction between downstream proteins; the middle nucleotide-binding oligomerization domain (NACHT, including NAIP, CIITA, HET-E and TP1 proteins) mainly mediate self-oligomerization; the leucine-rich repeats (LRRs) at the carbon end is mainly involved in recognition stimulation. LRR and NOD are ligand recognition domain and self-aggregation domain, respectively (Harijith et al., 2014). (Shao et al., 2015) Under normal physiological conditions, the NACHT domain of NLRP3 binds to LRRs, making it in a self-inhibiting state. When NLRP3 recognizes pathogens and intracellular danger signals, it undergoes oligomerization, binding to the adaptor protein ASC and recruiting caspase-1 precursors. These form the aggregates of the NLRP3 inflammasome. Caspase-1 aggregation and autocatalysis are induced. Inflammatory factors such as IL-1β and IL-18 are eventually activated by cleavage from inactive precursor forms and released in the extracellular space (Shao et al., 2015) (Figure 2).

ASC is an adaptor protein with a relative molecular mass of about 22,000, composed of PYD and CARD. ASC is mainly distributed in the nucleus of human monocytes/macrophages, it quickly transfers to the cytoplasm under stress, connects NLRP3 and proCaspase-1, and promotes the activation of NLRP3 inflammasome. NLRP3 and ASC bind by interacting with PYD-PYD and ASC binds with Pro-Caspase-1 by interacting with CARD (Figure 2), eventually forming inflammasome and activating inflammatory responses (Ye et al., 2017a).

Caspase 1 has pro-inflammatory effects in both mice and humans, and its catalytic activity is strictly regulated by inflammatory bodies. Caspase 1 can mediate the action of cytokines, such as IL-1β and IL-18 (Yan et al., 2015). Caspase-1, also known as IL-1β converting enzyme, is the effector protein of NLRP3 inflammasome. It is cleaved by the precursor molecule pro-Caspase-1 itself to form active Caspase-1, namely the two subunits of p20 and p10. Activated caspase-1 processes and modifies the precursors of pro-IL-1β and other inflammatory factors to promote their maturation and secretion, its powerful pro-inflammatory effect can directly affect the host’s innate immune regulation process against infection and injury (Sutterwala et al., 2014a).

## Activation of NLRP3 Inflammasome

### Classical Activation Pathway of NLRP3

The classical activation of the NLRP3 inflammatory complex generally requires two steps, including the preexcitation of NLRP3 inflammasome and activation of NLRP3 inflammasome (Liu et al., 2016a): 1) Initiation signal: NLRP3 is stimulated by the danger signal to initiate the transcription and expression of pro-IL-1β and NLRP3 and other precursor proteins through the nuclear factor κB pathway (Luo et al., 2014); 2) Activation signal: It is mainly the assembly of NLRP3 inflammasome and the process by which activated caspase-1 processes and modifies downstream inflammatory factors and finally leads to their mature secretion (Hoyt et al., 2017; Próchnicki and Latz, 2017). There are two primary forms of preexcitation: one is a transcription-dependent NLRP3 preexcitation. When NLRP3 agonists are used to directly stimulate macrophages, NLRP3 inflammasomes are not activated or only a little activated, but after pretreatment with bacterial ligands, the activation of NLRP3 inflammasomes will be significantly enhanced, this pretreatment process is called the initiation step, which provides the first signal for the activation of the NLRP3 inflammasome. In the absence of a start-up step, macrophages can express NLRP3 highly through retroviruses, and then given agonist stimulation can also enhance the activation of inflammasomes (Schroder et al., 2012). The study found that the activated macrophages could not induce the expression of NLRP3 protein in a short period of time (10 min), but significantly enhanced the activation of inflammasomes (Juliana et al., 2012; Schroder et al., 2012). It can be seen that the regulation of inflammasome activation during the initiation phase not only depends on transcriptional regulation, but also on post-transcriptional modification. While the other is activated by de-ubiquitination of the NLRP3 LRR region (de Rivero Vaccari et al., 2014). In the start-up phase, the coiled-coil structure of the carbon end of E3 ubiquitin ligase TRIM31 binds to the PYD domain of NLRP3, which promotes the K48-linked polyubiquitination of the NLRP3 protein and the degradation of the proteasome, thereby inhibiting the activation of the NLRP3 inflammasome (Song et al., 2016).

### Non-Classical Activation Pathway of NLRP3

In addition to classic NLRP3 inflammasome activation, there are also non-canonical NLRP3 acti vation pathways that rely on caspase 11, especially Gram-negative bacteria activate Toll-like receptor 4-myeloid differentiation factor 88 and β-interferon TIR domain adaptor protein path, at the same time, nuclear factor kappa B nuclear translocation occurs, thereby up-regulating the expression of NLRP3 inflammasome components, IL-1β and IL-18, and promoting the transcription of interferon regulatory factor 3 and interferon regulatory factor 7 genes (Latz et al., 2013). Activated caspase 11 does not directly cleave pro-inflammatory cytokine precursors, but activates the NLRP3-ASC-caspase 1 pathway through an unknown mechanism to promote the processing and release of IL-1β (Yi, 2017).

Classical and non-classical NLRP3 inflammasome activation pathways occur independently. Caspase 11 can enhance the activation process of classical pathway caspase 1 under specific stimulating conditions, and promote the production of IL-1β and IL-18 (Liu and Lieberman, 2017). Further *in vitro* experiments are needed to study the molecular mechanism of the interaction between caspase 1 and caspase 11 and the mechanism of caspase 11 activation of non-classical pathways or indirect activation of the classic NLRP3 inflammasome pathway.

### Regulation of Activation

#### Mitochondrial Dysfunction and NLRP3 Inflammasome Activation

Early studies have found that mitochondrial disturbance can promote the activation of NLRP3 inflammasome, and it is speculated that mitochondrial dysfunction is related to NLRP3 activation (Zhou et al., 2011). Mitochondria-associated adaptor, namely mitochondrial antiviral signaling protein (MAVS), are also related to the activation of NLRP3 inflammasome, but the specific mechanism of action is still controversial (Subramanian et al., 2013; Allam et al., 2014). Studies have shown that MAVS can interact with NLRP3, and it is necessary when soluble stimulants (such as ATP, nigericin) induce NLRP3 activation, and it is not necessary when stimulated by particulate matter [such as monosodium urate (MSU), alum] (Subramanian et al., 2013). Other studies have shown that MAVS plays a role in the activation of NLRP3 induced by RNA viruses, and does not play a role when induced by non-viral stimuli (such as ATP, nigericin) (Allam et al., 2014; Ermler et al., 2014). All in all, the exact role of mitochondria in the activation of NLRP3 inflammasomes is still unclear. Mitochondrial derived reactive oxygen species (ROS) are key signals regulating NLRP3 inflammasome activation (Liu et al., 2016a). As a product of mitochondria, mitochondrial reactive oxygen species (mROS) activates NLRP3 inflammasomes and serve as a secondary signal for the formation of mature IL-1β (Sutterwala et al., 2014b). Activation of NLRP3 inflammasomes has been observed by disrupting mitochondrial function with chemical inhibitors (de Lavera et al., 2017).

#### K^+^ and NLRP3 Inflammasome Activation

Activation of NLRP3 requires K^+^ efflux in the non-classical inflammatory body pathway (Genipin suppresses3, 2015). Studies have also shown that a decrease in the concentration of intracellular potassium has an activating effect on the NLRP3 inflammasome, and can cause the activation of caspase-1 dependent on the NLPR3 inflammasome (Patel et al., 2017). Other studies have shown that low concentrations of K^+^ can also cause the aggregation of NLRP3 inflammasomes in a cell-free system (Pétrilli et al., 2007). Therefore, related studies have shown that the decrease of intracellular K^+^ concentration is considered to be the common mechanism of NLRP3 inflammasome activation (Muñoz-Planillo et al., 2013). In mutated macrophages with no potassium outflow, NLRP3 inflammatory bodies undergo activation and are not affected by extracellular high potassium ion concentrations (Zhou et al., 2011). This indicates that the decrease of K^+^ concentration in the cell may cause the conformational change of NLRP3, and this change is consistent with the conformational change caused by NLRP3 activating mutation.

#### Activation of Particulate Matter and NLRP3 Inflammasome

The particulate matter destroys the lysosomal membrane through endocytosis, resulting in the release of cathepsin B, a lysosomal cysteine protease of the papain family, into the cytoplasm and activates the NLRP3 inflammasome (Amaral et al., 2018). Several studies have shown that treating macrophages with CA-074-Me, a chemical inhibitor of cathepsin B, can inhibit the activation of NLRP3, further studies have found that after stimulating macrophages lacking cathepsin B with particulate matter, the activity of NLRP3 has no effect (He et al., 2016a), this indicates that cathepsin B inhibitors inhibit the activation of NLRP3 inflammasomes may be due to non-target effects or redundancy among cathepsin family members, the conclusion that echoes the above conclusion is that cathepsin, which can be inhibited by CA-074-Me inhibitor, promotes the priming and activation process of NLRP3 (Orlowski et al., 2017). In addition to releasing cathepsin to the cytoplasm and activating NLRP3 inflammation, other factors can also trigger cell K^+^ efflux and activate NLRP3 inflammatory bodies (Shil et al., 2018). The lysosomal inhibitor leu-leu-o-methyl ester was found to induce rapid K^+^ efflux in macrophages before NLRP3 inflammasome activation (Liu et al., 2016b).

#### Ca^2+^ Signal and NLRP3 Inflammasome Activation

Studies have found that the Ca^2+^ chelating agent 1, 2-bis (2-aminophenoxy) ethane-N, N, N′, N′-tetraacetic acid tetraacetoxymethyl ester (BAPTA-AM) can inhibit IL-1β Secretion, therefore, it is speculated that Ca2+ signal may be involved in the activation of NLRP3 inflammasome (Chu et al., 2009). NLRP3 inflammasome requires the participation of intracellular calcium ions for the maturation and release of IL-1β, depleting calcium ions in the endoplasmic reticulum or inhibiting the influx of extracellular calcium ions can effectively inhibit the activation of NLRP3 inflammasome, and can reduce the formation of IL-1β (Ystgaard et al., 2015). It is worth noting that Ca^2+^ mobilization and mitochondrial calcium overload may cause mitochondrial dysfunction, which in turn promotes the activation of NLRP3, the addition of Ca^2+^ to the extracellular medium will cause the formation of particulate matter and K^+^ outflow, which will complicate these experimental results (Muñoz-Planillo et al., 2013).

#### Other Mechanisms

MicroRNA also plays an important role in inflammation. miR-7, which regulates the synthesis of α-synuclein, is not only expressed in dopaminergic neurons (Lee et al., 2018), but also in microglia to inhibit the occurrence of inflammation (Chen et al., 2018). MiR-29c-3p (miR-29c) has anti-inflammatory effects in PD animals and neuronal models, and can target activated nuclear factor 5 of activated T cells (NFAT5) to regulate the NLRP3 inflammasome (Zhang et al., 2019). MiR-190 has also been shown to reduce neuronal damage and inhibit inflammation by negatively regulating the expression of NLRP3 in a MPTP-induced PD-model (Wang et al., 2020). MiR-135b can target the transcription factor forkhead box O1(FoxO1) to inhibit NLRP3 inflammasome and pyrolysis, and play a protective role in PD (Sun et al., 2019). MiR-30e can directly target NLRP3, inhibit the expression of NLRP3, and improve the occurrence of neuroinflammation (Zeng et al., 2019). The occurrence of PD is closely related to the NLRP3 inflammasome, and microRNA may become the target of PD treatment. In addition, studies have found that in human and mouse cells, the application of drugs to inhibit the ubiquitination of NLRP3 inflammasomes can also inhibit the activation of human and mouse NLRP3 inflammasomes (Li et al., 2018). However, ubiquitin has a wide range of effects. Using ubiquitin as a target to inhibit the formation of NLRP3 inflammasomes, its specificity is a major challenge.

### Signaling Molecules Involved in the Regulation of NLRP3 Inflammasome Activation

Irritants inducing NLRP3 inflammasome activation depend on Nek7, such as ATP, nigrotin, microcrystalline sodium urate crystals and alum (Cheng et al., 2020). The catalytic region of Nek7 and the LRRs region of NLRP3 can interact to form a NLRP3-Nek7 macromolecular complex, NLRP3 agonists can enhance this effect, and Nek7-mediated activation of NLRP3 has nothing to do with its kinase activity (He et al., 2016b; Shi et al., 2016). Nek7 controls NLRP3 inflammasome activation and ASC half-point formation leading to intracellular K^+^ outflow (Shi et al., 2016). Nek7 has been shown to be a key link in the activation of NLRP3 inflammasome. Moreover, the absence of Nek7 reduces the secretion of IL-1β and the recruitment of immune cells (He et al., 2016b). These results all indicate that Nek7 is a positive regulator of NLRP3 inflammasome activation. When there is a high concentration of K^+^ outside the cell, the interaction between Nek7 and NLRP3 is inhibited, indicating that this interaction requires the outflow of K^+^, it is considered that the decrease in K^+^ concentration in the cell causes the conformational change of NLRP3, which promotes the binding of Nek7 to NLRP3; when NLRP3 has an activating mutation that does not depend on K^+^ efflux (NLRP3^R258W^), the activation of inflammasomes still requires Nek7 (He et al., 2016b). Therefore, exploring the response mechanism of Nek7 to NLRP3 agonists will provide new ideas for the study of the molecular mechanism of NLRP3 inflammasome activation.

## NLRP3 Inflammasome and Depression

In today’s fast-paced society, the incidence of depression is worryingly increasing every year, with a current global incidence of about 20% (Wang et al., 2014a). According to the World Health Organization, depression in 2020 will become the second largest human health burden after cardiovascular diseases, causing an economic burden of approximately US$2.5 trillion, accounting for 10% of the global disease burden (Tran et al., 2020).

At present, there are many theories to explain the pathogenesis of depression. These include neurotransmitter dysfunctions, endocrine disorders, damage to neuronal adaptability and plasticity, oxidative stress and mitochondrial dysfunction, amongst others (Ogodek et al., 2014). However, all these theories can only partially explain the development of depression. Recent studies have shown that immune system activation and cytokine levels might mediate the development of depression (Zhang et al., 2014). Interestingly, it has been reported that the levels of pro-inflammatory factors, cytokines and related receptors in peripheral blood of patients with depression are higher than normal (Miller and Raison, 2016). Levels’ of inflammatory factors are closely related to the severity of depression. Additionally, inhibiting inflammatory signaling pathways can reduce patients’ related emotional symptoms of depression (Köhler et al., 2014).

Over the past decade, there have been several studies investigating the relationship between cytokine abnormalities and depression. From the peripheral and central immune system perspective, many studies from clinical and preclinical models have provided conclusive evidence of a link between cytokine abnormalities and depression (Köhler et al., 2014; Miller and Raison, 2016). Currently, known cytokines associated with depression are IL-1β, IL-6, TNF-α, IFN-γ, c-reactive protein (Müller, 2014). The most widely studied are IL-1β, IL-6 and TNF-α, and their roles are relatively understood.

Chronic stress can activate nod-like receptors (NLRs) family member NLRP3, which can be connected with apoptosis-associated speck-like protein containing CARD. Thus, pro-cysteinyl aspartate specific proteinase 1 (pro-caspase-1) containing cysteine can be converted into activated caspase-1, which further promotes the secretion of interleukin-1 (IL-1). This induces an inflammatory response which might participates in the pathogenesis of depression (Pan et al., 2014; Zhang et al., 2014). This connection sheds light on the physiological causes of depression and provides a new target for potential treatments.

### Innate Immunity and Depression

Recently, Kéri et al. (2014) found that high level of TLR4 in monocytes of depressed patients. Following antidepressant treatment, the depressive symptoms improved and also the levels of TLR4 significantly decreased, further indicating that TLR4 might be correlated with the pathology of depression. Further studies (Liu et al., 2014) showed that the role of TLR4 in depression may be related to the interaction between TLR4 and the hypothalamus-pituitary-adrenal cortex (HPA) axis. The activation of TLR4 can, in fact, affect the activity of the HPA axis both in the short and long term, under the action of chronic stressors. This conclusion is consistent with previous findings (Kéri et al., 2014) and further supports the important relationship between TLR4 activity and the pathological process of depression.

### The Link Between NLRP3 Inflammasome and Depression

Recently, a lot of research effort has been put in the study of immune inflammation, both in China and worldwide. Immune inflammation has always been closely related to depression (Liu et al., 2014). An appropriate animal model of immune inflammation is an essential tool to explore the immune inflammation hypothesis of depression. Although Chronic unpredictable mild stress (CUMS) remains the most common model of depression in basic research, LPS-induced animal models of depression are being gradually recognized and accepted (Slavich and Irwin, 2014). LPS induces the release of oxidative stress and pro-inflammatory factors, leading to corticotrophin-releasing hormone (CCH), elevated serum corticosterone, dysfunction of the hypothalamic-pituitary-adrenal axis, and depression-like behavior in animals (Honokiol abrogates lipopo, 2014). It has been suggested that LPS-induced activation of the NLRP3 inflammasome leads to the release of IL-1 and IL-18 (Leff-Gelman et al., 2016). After NLRP3 is activated, it connects with caspase-1 through ASC to release IL-1, TNF-a, NO and ROS by activating indoleamine 2, 3-dioxygenase (IDO). This causes a decrease in serotonin in the brain and induces neuronal cell apoptosis (Alcocer-Gómez et al., 2014). In addition, mice with the NLRP3 gene knocked out did not exhibit depression-like behavior after prolonged CUMS stimulation (Alcocer-Gómez et al., 2016), suggesting that NLRP3 inflammasome is closely associated with depression.

The course of depression is often associated with changes in pro-inflammatory substances such as NLRP3 and IL-1β (Alcocer-Gómez, 2014). Animal models showed that after entering the brain, inflammatory mediators affect various neurotransmission mechanisms such as glutamate release, microglia and astrocytes uptake, and production of neuroactive metabolites. These effects increase nerve excitability, leading to oedema, depression and neuroinflammation (Kaufmann et al., 2017). A clinical study (Choi and Ryter, 2014) showed that NLRP3 inflammatory body, IL-1β and IL-18 expression in brain tissue and serum of depressed patients were significantly higher than normal. Research showed that in the LPS-induced depression murine model, following LPS treatment for 24 h, NLRP3 brain inflammation, ASC and caspase 1 and IL-1β mRNA protein levels increased significantly. The caspase 1 inhibitor Ac-YVAD-CMK can inhibit the activation of NLRP3 and the inflammation induced by LPS, indicating that NLRP3 inflammasome and caspase 1 are involved in the depression model induced by LPS (Zhang et al., 2014). Liu et al. (2015) supported this conclusion. They found that the hippocampal region NLRP3 inflammatory body was also significantly activated in the depressed mouse model. Specific inflammasome inhibition of vx-765 inhibited NLRP3 inflammasome activation in the hippocampus of mice induced by chronic unpredictability and mild stress and improved depression-like behavior in mice (Alcocer-Gómez et al., 2016). No increase in IL-1β levels, microglial activation, or hippocampal neurogenesis were observed in NLRP3 knockout mice (Xu et al., 2016). The inflammasome inhibitor vx-765 reversed elevated levels of IL-1 and IL-18 in the hippocampus and improved depression-like behavior in these models of depression (Zhang et al., 2015a). In addition to these findings, no increase in IL-1β, microglial activation, and decreased hippocampal neurons were observed in NLRP3 mice that were knocked out of the inflammatory corosome gene. Additionally, no studies were performed on a stress-induced mouse model of depression (Alcocer-Gómez et al., 2016; Iwata et al., 2016).

### The Relationship Between IL-1β, an Inflammatory Body Activation Marker of NLRP3, and Depression

IL-1β activates the HPA axis and induces depression-like behavior in rats. IL-1β represents the initial step in the pro-inflammatory response to psychological stress, leading to cell damage in stress-related diseases, including depression (Alcocer-Gómez et al., 2016). Elevated levels of IL-1β in subjects with childhood maltreatment and other stressors are closely associated with depression-like behavior in brain tissue and serum (Hyun-Jung et al., 2015). A polymorphism in the IL-1β gene C511T, which did not differ in genotype or allele distribution between patients and controls, was reported. However, individuals with homozygous C511T allele showed increased chances of developing depression, were less responsive to fluoxetine and paroxetine treatment, and had a younger onset of depression in the elderly (Tartter et al., 2015).

### Role of NLRP3 Inflammasome in the Pathogenesis of Depression

#### NLRP3 Inflammasome and Peripheral Immunity in Depression

Numerous studies (Müller, 2014) have confirmed that inflammatory factors play an important role in the pathogenesis of depression. The first evidence that the NLRP3 inflammasome might be involved in the pathogenesis of depression came from behavioral studies in a mouse model of lipopolysaccharide-induced depression (Zhang et al., 2014). The study also found that NLRP3 inflammasome inhibitors block depressive behavior. Zhang et al. (2014) also demonstrated that increased NLRP3 inflammatory body expression can increase serum IL-1β. Alcocer-Gomez (Alcocer-Gómez et al., 2014) studied 20 patients with untreated depression undergoing amitriptyline treatment and 20 healthy controls. They compared peripheral blood mononuclear cells in the NLRP3 inflammasome and caspase 1 gene expression. They found that in the depression group, there were more activated peripheral blood corpuscle and NLRP3 inflammation compared to the control group. Moreover, in the amitriptyline treatment group, the NLRP3 inflammasome was reduced. A clinical study (Alcocer-Gómez et al., 2014) found a high correlation between NLRP3 activation and IL-1β/IL-18. In addition, antidepressant drugs have been reported to reduce serum IL-1β levels without lowering tumor necrosis factor-α (TNF-α) levels, further suggesting that IL-1β responds to anti-depression therapy. The results of Zhang et al. (2015a) in the animal model confirm this theory. They also found that the increased expression of each component of NLRP3 inflammasome and the enhanced activity of the inflammasome can increase the level of serum IL-1β. Recently, Su et al. (2017) found that the classic animal model of chronic mild unpredictability stress could not successfully establish an animal model of depression when the NLRP3 gene was knocked out, and that the level of IL-1β was not increased in peripheral blood.

#### NLRP3 Inflammasome and Central Immunity in Depression

In the central nervous system, the activation of NLRP3 inflammasome in the microglia can impair the blood-brain barrier’s permeability and integrity. This enables peripheral immune cells to enter the central nervous system and participate in intracerebral inflammatory response through active transport, vagus nerve stimulation, destruction of vascular endothelial cells and other pathways (Wohleb et al., 2016). Research by Bufalino et al. (2013) found that there are a large number of endogenous immune factors, such as IL-1β, IL-6, TNF-α, TLR3 and TLR4, in brain samples of depression patients who died by suicide. The hippocampus also plays a vital role in the onset of depression, and IL-1β inhibits the formation of hippocampal neurons, thereby mediating the occurrence of depression (Réus et al., 2015). Iwata et al. (2016) also confirmed this conclusion. They found that NLRP3 inflammatory body was significantly activated in the hippocampus of depressed mouse models. The activated inflammatory body could directly damage the regeneration of nerve cells. Early studies (Goshen et al., 2008) of animal models of depression have found that IL-1β mediates depression by inhibiting the formation of hippocampal neural precursor cells and the activation of adrenal cortex function. Recently, Liu et al. (2015) found that NLRP3 was activated in the hippocampus using in an animal model of depression, consistent with previous results and further explaining why IL-1β levels were increased in depression. The mice were also more tolerant to learned helplessness. Iwata et al. (2016), using the NLRP3 gene knockout mouse model, found that stress could change the activity of ATP-p2x7r-NLRP3 inflammatory signaling pathway in the hippocampus and induce depression. Alcocer-Gómez et al. (2016) found that in an animal model of chronic stress-induced depression, the expression of IL-1β mRNA and protein in the prefrontal cortex and hippocampus of mice were increased, which further proved that depression-related behaviors depended on the expression of NLRP3 gene.

### NLRP3 Inflammatory Body as a New Potential Target for the Treatment of Depression

Hannestad and others (Hannestad et al., 2011) found that anti-inflammatory drugs also have antidepressant effects, so does antidepressant treatment have anti-inflammatory effects? The basic and clinical research for depression has found that NLRP3 inflammasomes are in an activated state, and antidepressant treatment can inhibit the activity (Xue et al., 2015) of NLRP3 inflammasomes. Significant changes in NLRP3 were found in both central and peripheral studies of animal depression models (Su et al., 2017), suggesting that NLRP3 inflammasomes may be an important target for depression treatment. From basic and clinical research in the pathogenesis of depression (Alcocer-Gómez et al., 2014), it is clear that, in depression, there are higher levels of peripheral blood mononuclear cells of NLRP3 inflammasome product components. Moreover, the effect of IL-1β is significantly higher. NLRP3 inflammasome will gradually return to normal after antidepressant treatment. Antidepressant treatment inhibits NLRP3 inflammasome (Xue et al., 2015), highlighting the relationship between depression and NLRP3. Amittiline, a tricyclic antidepressant, inhibited NLRP3 and caspase-1 gene expression and reduced serum IL-1β/IL-18 levels. However, no correlation between mitochondrial reactive oxygen species products and inflammatory body activation was found (Alcocer-Gómez et al., 2014). At the same time, Abbasi et al. argued that mitochondrial dysfunction is not involved in the activation of inflammatory bodies in depression, in disagreement with the previous conclusion (Alcocer-Gómez et al., 2014).

As the first-line clinical antidepressant treatment of selective serotonin reuptake inhibitor (SSRI) drugs, whether their antidepressant treatment affects the activity of NLRP3 inflammasome is also a question that needs to be clarified. Fluoxetine could reduce the production of ATP-induced ROS and the phosphorylation of double-stranded RNA-dependent protein kinase (PKR) in primary cultured macrophages and microglia *in vitro*. In this study, the link between PKR and NLRP3 inflammatory body was not present, and the activation of NLRP3 inflammatory body was inhibited (Du et al., 2016). Du et al. (2016) found that fluoxetine can significantly inhibit NLRP3 inflammatory body activation, caspase-1 cleavage and IL-1β secretion in peripheral macrophages and central microglia. Fluoxetine can also significantly reduce the production of reactive oxygen species. A recent study by Pan et al. (2014) found that fluoxetine reverses NLRP3 inflammasome formation and IL-1β levels in the prefrontal cortex. The above research results show that the anti-inflammatory mechanism of SSRI and tricyclic antidepressants may be different, but both drugs can have a certain effect on the NLRP3 inflammasome. So whether the NLRP3 inflammasome is a central target of antidepressant therapy needs to be further clarified. Although the specific mechanism of NLRP3 inflammasome in antidepressant treatment is still unexplained, it can be speculated that it can be used as one of the potential biomarkers to evaluate the effect of antidepressant treatment.

In addition, l-menthone and thymol increased serotonin and norepinephrine levels in the hippocampus of mice treated with chronic stress, reduced IL-1β levels, and inhibited the activation of NLRP3 inflammatory bodies in the hippocampus of mice (Deng et al., 2015a). Apigenin, a natural flavonoid, inhibits NLRP3 inflammatory particles’ activation and increased IL-1β levels in the prefrontal cortex of rats caused by chronic and unpredictable mild stress through PPAR-γ receptors (Li et al., 2016). Nonannular monoterpene geranyl ACTS as an antidepressant and anti-inflammatory by reducing the expression of NLRP3 inflammatory-associated proteins and their downstream target gene IL-1β through the nf-κ B signaling pathway (Deng et al., 2015b). Butylamine is an antidepressant polyamine that significantly down-regulates the expression of NLRP3 inflammasome components in the hippocampus and prefrontal cortex of subchronic stress-treated rats, including caspase-1, IL-1β, and IL-18 (Sharon et al., 2016). Therefore, the NLRP3 inflammasome may become a new target for improving depression in the future.

## NLRP3 Inflammasome and AD

AD is a common chronic, multifactorial degenerative disease of the central nervous system occurring in the elderly. AD’s clinical features include progressive memory loss, cognitive impairment and personality changes, which place a tremendous burden on individuals, families and society (Zuroff et al., 2017; Gao et al., 2018). The deposition of amyloid-protein (Ap) in the brain is thought to cause chronic inflammation and secondary neuronal death (Choi and Ryter, 2014). The expression levels of interleukin IL-1 and IL-18 of microglia (MG) in the brain of AD patients are significantly upregulated. In AD mouse models, the specific blocking of IL-1β could slow down Tau protein’s hyperphosphorylation in the brain and rescue the cognitive impairment (Díaz et al., 2014).

### Inflammasome and AD

The inflammatory response is mediated by pro-inflammatory factors and produces a persistent chronic inflammatory response between activated glial cells, stressed neurons, and Aβ plaques (Heppner et al., 2015). NLRP1 silencing plasmid intervention in APP/SP1 transgenic mice reduced caspase-1 expression, reduced apoptosis and improved the mice’s cognitive function. NLRP1/caspase-1 signaling pathway may be related to the onset of AD (Tan et al., 2014). Glucocorticoid impairment of learning and memory can accelerate the progression of AD disease. Studies have shown that the expression of hippocampal neurons NLRP1, caspase-1, IL-1β and IL-18 increase with behavioral changes after long-term exposure of glucocorticoid to mice (Yaodong et al., 2017). As active products of NLRP3 inflammasome, IL-1β and IL-18 are at the upper end of the inflammatory chain in the neuronal inflammatory response of AD and play an important role in initiating and maintaining the inflammatory response (Ben Menachem-Zidon et al., 2015). IL-1β can work with TNF- a and other inflammatory factors to damage neurons and inhibit long-term potentiation (LTP), thereby affecting learning and memory functions (Lynch and Marina, 2015). In addition, meta-analysis studies showed that gene polymorphism in the human IL-18 promoter region is correlated with the risk and prognosis of AD (Jiaojiao et al., 2016). More studies have shown increased expression of inflammosome-related proteins in brain tissues of patients with AD, including NLRP1, which has been confirmed to be associated with AD gene variation. Also, increased levels of NLRP1 were found in the brain of APPswe/PS1dE9 transgenic mice and increased expression of NLRP1/caspase-1 signaling pathway in cortical neurons induced by Aβ *in vitro* experiment leading to apoptosis of cortical neurons (White et al., 2017). Neuronal inflammatory response induced by NRLP1 inflammasome plays an important role in AD’s pathogenesis. NRLP1 may induce downstream caspase-1 expression to release pro-inflammatory factors IL-1β and IL-18 (Hu et al., 2016; Zhang et al., 2017a) by activating the neuronal NLRP1 inflammasome. The specific mechanism may be related to the opening of potassium channel (Zhang et al., 2017b).

AD patients with brain autopsy results confirmed that NLRP3 gene mutations increase the risk of late-onset of dementia. The study found that in patients with moderate and severe AD, NLRP1, NLRP3, caspase 1 and its downstream material IL-1β and IL-18 expression increased, together with prompt activation of the inflammasome closely associated with AD nerve inflammation (Saresella et al., 2016). Another study using the APP/long-term SP1 transgenic mice found that mitochondrial oxidative damage and severe brain inflammation, are accompanied by inflammasome NLRP3 activation. Interestingly, NLRP3 expression of hydrogen peroxide reductase expression in transgenic mice also increased, and the group of mice with brain nerve inflammation had aggravated cognitive impairment (Chen et al., 2015). The activation of NLRP3 phagocyte NADPH oxidase can lead to the generation of reactive oxygen species (Choi and Ryter, 2014). Aβ stimulates microglia to produce reactive oxygen species via mitochondria and NAPDH oxidase, which can also activate NLRP3 inflammasomes to produce caspase-1 and increase the neurotoxicity of microglia (Parajuli et al., 2013). These results suggest that oxidative stress is related to AD. Oxidative stress may trigger neuroinflammatory response by activating inflammasomes, leading to abnormal synaptic function and neuron loss. Oxidative stress produces reactive oxygen species that may act as a signal molecule to initiate cell pyrolysis, induce neuroinflammation and neuronal death. Therefore, antioxidants may inhibit the activation of inflammasomes, reduce the production of pyroptosis and play an important role in the prevention and treatment of AD.

### NLRP3 Inflammasome May be a Therapeutic Target for AD

Studies have shown that citreolide can inhibit the activation of various inflammasomees of macrophages by directly reducing the activity of caspase-1 protease (Huai et al., 2014). Studies have shown (Dempsey et al., 2017) that the use of NLRP3 inhibitors can reduce APP/PS1 transgenic mice amyloid deposition, improve cognitive function, and inhibit microglial cell activation and inflammatory body activation in transgenic mice, thereby inhibiting the occurrence of pyroptosis. Recent studies have shown that ornidazole inhibits NLRP3 inflammasome in mice by reducing ROS production and suppressing NF-κB activity (Ye et al., 2016). Further studies have shown that inhibition of NLRP3 inflammasomees is independent of their effect on NF-κB activity (Wang et al., 2015a). It is suggested that their inhibitory effect may be partially realized through their inhibition of NLRP3 ATPase activity (Wang et al., 2015b). In addition, NLRP3 inhibitors have been reported include type 1 interferon (IFN-α and IFN-β), and they are used as a treatment of multiple sclerosis, juvenile idiopathic arthritis, rheumatic diseases, familial Mediterranean fever and other autoimmune diseases caused by mutations in NLRP3 gain of function, but the specific mechanism of their NLRP3 still needs to be studied in depth (Youm et al., 2015a; Malhotra et al., 2015). NLRP3 mutations can lead to juvenile idiopathic arthritis, rheumatoid arthritis and autoimmune diseases such as familial Mediterranean fever clinical commonly used drugs (Youm et al., 2015b; Malhotra et al., 2015). CRID3, a cysteine leukotriene receptor antagonist, was also found to down-regulate NLRP3 inflammasome activity by inhibiting ASC aggregation in mouse macrophages (Coll et al., 2015), but the specific mechanism remains to be investigated in the context of NLRP3.

## Role of NLRP3 Inflammasome and Ischemic Stroke

Ischemic stroke is the second leading cause of death worldwide and the leading cause of permanent disability in adults (Ye et al., 2017a).

NLRP3 inflammasome can lead to ischemic stroke by activating inflammatory response and participating in atherosclerosis (Hoseini et al., 2018). The expression of NLRP3 inflammasome was increased in the *in vitro* neuronal OGD model and the vector mouse cerebral ischemia model. After using the NLRP3 inhibitor MCC950, the expression of NLRP3 inflammasome-related protein and activated caspase-3 were reduced. However, the expression of P2X7 receptor did not change significantly, suggesting that NIRP3 inflammasome also plays an important role in neuronal apoptosis after cerebral ischemia (Ye et al., 2017a). After cerebral ischemia, a series of pathophysiological changes and the stimulation of danger signals can activate the inflammatory body. NLRP3 inflammatory body can sense the danger signals inside and outside the cell, for example, an increase of extracellular ATP, a decrease of intracellular K^+^ concentration caused by K^+^ outflow, the production of ROS in large amount and the destruction of lysosomes (Keren et al., 2016). After Ischemia/reperfusion (I/R) injury, NLRP3 deficiency improved neurovascular injury, reduced infarct size, and attenuated neurofunctional deficits (Zhang et al., 2015b). BTK inhibitors can inhibit the activation of NLRP3 inflammasome, reducing the volume of cerebral infarction, and improving the neurological impairment after I/R injury (Ito et al., 2015).

### NLRP3 Inflammasome in the Treatment of Ischemic Stroke

#### Sinomenine

**FIGURE 3:**
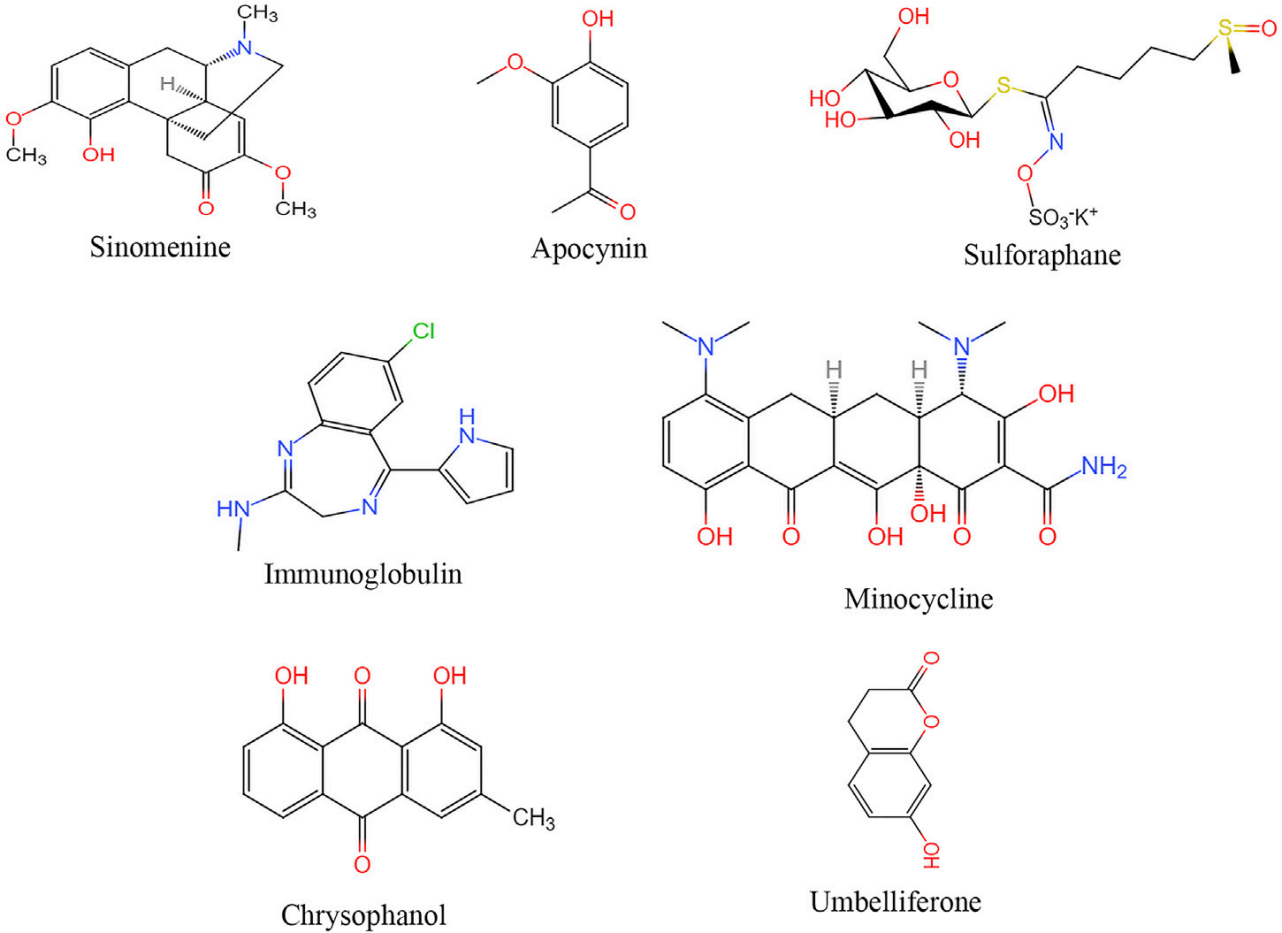
Structural of drugs targeting NLRP3 for treatment ischemic stroke.

(Figure 3) Nowadays, a wide range of pharmacological and clinical research on Sinomenine concentrates on the immune response, cancer, cardiovascular disease and neural system disease. Sinomenine’s action mechanism may inhibit macrophage, peripheral blood mononuclear cells and microglia activation, reduce prostaglandins E3, TNF-α, IL-1β, IL-6 and IL-1 factor secretion and regulating immune responses (Zhang et al., 2015c). Sinomenine also reduces mtROS and ATP production after I/R injury and inhibited NLRP3 inflammasome activation by inhibiting AMPK pathway (Qiu et al., 2016).

#### Irisin

Irisin can regulate the expression of NLRP3 after I/R injury, reduce or inhibit oxidative stress and inflammatory response (Peng et al., 2017). Irisin may alleviate neuronal injury caused by oxygen and sugar deprivation by inhibiting the ROS-NLRP3 signaling pathway (Peng et al., 2017).

#### Apocynin

Both NADPH and Apocynin inhibit the expression of NLRP3, ASC, caspase-1, IL-1β, and IL-18 in the ischemic cerebral cortex in a rat cerebral infarction model, and reduce the volume of cerebral infarction, improve survival after infarction, and restore neurological function (Qin et al., 2018).

#### Nuclear Factor E2-Related Factor-2 Related Drugs

Studies have shown that Nrf2 can inhibit the formation of NLRP3 inflammasomes through thioredoxin-1 (Trx1)/thioredoxin interacting protein (TXNIP) during cerebral ischemia-reperfusion injury, thereby playing a protective role in the brain. TXNIP is separated from the Trx1/TXNIP complex and is closely related to the activation of NLRP3. In the mouse middle cerebral artery occlusion model, the expressions of TXNIP, NLRP3, caspase-1, IL-18 and IL-1β were significantly reduced after treatment with tert-butyl hydroquinone up-regulated Nrf2, while knocking out Nrf2 and Trx1 was the opposite, it also inhibited the protective effect of Nrf2 when Trx1 was knocked out. In patients with liver injury and severe lupus nephritis, Nrf2 can promote the activation of antioxidant response element (ARE) genes and inhibit the activation of NLRP3 inflammasome. This study suggests that Nrf2 inhibits NLRP3 inflammasome activation during cerebral ischemia/reperfusion injury by regulating the Trx1/TXNIP complex (Hou et al., 2018).

Before the oxygen glucose stripping reperfusion model was established, BV2 cells were pretreated with tert-butyl hydroquinone or Nrf2 CRISPR plasmid. Nrf2 activation can inhibit the expression of NLRP3 inflammasome and the formation of downstream IL-1β. Moreover, the activation of NLRP3 inflammasome is very sensitive to the level of ROS, and Nrf2 can reduce the production of ROS. In addition, as a gene-targeted by Nrf2 to ARE, NADPH quinone REDOX enzyme is involved in the inhibition of NLRP3 inflammasome (Xiujian et al., 2018). This provides a new therapeutic target for cerebral ischemia-reperfusion injury.

#### Sulforaphane

SFN treatment of rats with middle cerebral artery occlusion inhibited the activation of inflammatory vesicles and the expressions of caspase-1, IL-1β and IL-18, reduced the volume of cerebral infarction, improved the score of neurological function, and reduced neutrophil infiltration (Yu et al., 2017).

#### Other Therapeutic Approaches

Studies have suggested that intermittent fasting, intravenous immunoglobulin, minocycline, emodin, tyrosine kinase can all inhibit the activation of NLRP3 inflammasome in cerebral ischemia-reperfusion (Fann et al., 2014; Zhang et al., 2015b; Leff-Gelman et al., 2016; Lu et al., 2016). Umbel can inhibit TXNIP/NLRP3 inflammatory bodies by upregulating the expression of peroxisome proliferators-activated receptor (PPAR)-γ (Wang et al., 2015c). Transfected small interfering RNA (siRNA) of Sirt1 blocked the inhibition of autophagy activity by resveratrol and inhibited the activation of NLRP3 inflammasomes (He et al., 2017). Minocycline inhibited NLRP3 inflammatory bodies’ activation and associated inflammatory responses in mouse models, significantly improving brain injury induced by cerebral ischemia-reperfusion (Lu et al., 2016).

## NLRP3 Inflammasome and Intracranial Haemorrhage

Intracranial haemorrhage (ICH), including ICH and subarachnoid haemorrhage (SAH), has a high mortality and disability rate, accounting for 10–20% (Xi et al., 2013) of all types of stroke. On the one hand, abnormal or maladjusted inflammatory response after intracranial haemorrhage will aggravate cell damage in the damaged part of brain tissue (Wang et al., 2014b). On the other hand, the inflammatory response can remove apoptotic cells and cell debris, contributing to tissue reconstruction and repair (Schallner et al., 2015). In the central nervous system, the positive aspect of the inflammatory response is that immune cells can remove necrotic cells, support the barrier system, and create suitable conditions for wound healing. Therefore, those who think that the neuroinflammatory response after intracranial hemorrhage is completely harmful is not scientific.

For example, microglia are the most important immune regulatory cells in the central nervous system, and they are usually the first cells to respond after brain injury. In a meningeal contusion model, the ATP released by damaged astrocytes can cause the microglia in the deep part of the brain to turn into an activated state and remove the necrotic cell debris. Compared with the control group, inhibiting this response resulted in more neuronal apoptosis (Corps et al., 2015). After the white matter of the corpus callosum is damaged, inhibiting the activity of microglia can also slow down the removal of myelin debris, thereby inhibiting the accumulation and remyelination of oligodendrocyte precursor cells, aggravating axon damage (Lampron et al., 2015). In addition, microglia may also be involved in maintaining the stability of the blood-brain barrier after brain injury. For example, in the laser damage model, the capillary damage caused by the laser leads to a significant increase in the permeability of the blood-brain barrier. Microglia can quickly gather around the capillary wall and stretch out a large number of protrusions to wrap around, thereby preventing the leakage of the blood-brain barrier. Inhibiting this response of microglia can significantly increase the permeability of capillaries (Lou et al., 2016). Therefore, the key participants in the acute phase of the microglia central nervous system injury are not only to release pro-inflammatory factors, but also to provide barrier support and clean up debris, which has a certain beneficial effect.

In fact, microglia can be activated and transformed into M1 type (classical activation) and M2 type (alternative activation type) when they are stimulated by damage stimulating factors. Among them, M1 type microglia/macrophages can express IL-1β, IL-6, IL-12, IL-23, TNF-α, NOX2 and other cytokines that promote inflammation, and aggravate nerve function damage; M2 type microglia/macrophages can secrete anti-inflammatory factors such as IL-4, IL-10 and transforming growth factor-β (transforming growth factor-β, TGF-β), which can reduce neurological function after intracranial hemorrhage damage (Kumar et al., 2016). Therefore, we believe that inflammation promotes nerve damage after intracranial hemorrhage is related to the M1 polarization of microglia/macrophages. The M2 polarization of microglia/macrophages is essential for the reconstruction and repair of the central nervous system. The challenge we are currently facing in the anti-inflammatory treatment of intracranial hemorrhage is how to develop drugs to promote the transformation of the early inflammatory response after intracranial hemorrhage to a beneficial aspect, and to prevent neuroinflammation that damages nerve function.

### Role and Regulatory Mechanism of NLRP3 Inflammasome in Intracranial Haemorrhage

#### NLRP3 Inflammasome and ICH

ICH is a common disease in neurosurgery, accounting for about 10–15% of all types of cerebral apoplexy, with more than 200 million patients in the world each year. It has a high disability rate and fatality rate, resulting in a heavy burden to families and society (Al-Khaled and Eggers, 2014). Many studies have shown that neuronal injury caused by aseptic inflammatory response is a key factor leading to secondary injury after cerebral haemorrhage (Zhou et al., 2014). Infiltrating inflammatory cells release large amounts of inflammatory factors, free radicals, and other toxic substances that further aggravate nerve cell damage and affect patients’ prognosis (Yang et al., 2014). Microglia, which first appear around hematoma, are important immune cells of the central nervous system and mainly participate in the central nervous system’s innate immune response (Walsh et al., 2014).

Numerous studies have shown that aseptic inflammatory response after intracerebral haemorrhage plays an important role in secondary brain injury (Zhou et al., 2014). To date, researchers have demonstrated that large amounts of inflammatory cytokines, such as IL-1β, IL-6, TNF-α, can be detected around hematomas after intracerebral hemorrhage in humans and animals. IL-1β plays a key role in many inflammatory cytokines, participating in multiple cellular functions and promoting the synthesis and release of other inflammatory cytokines (Patterson, 2015). It has been demonstrated that NLRP3 activation and formation of NLRP3 inflammasome in microglia surrounding hematoma after intracerebral hemorrhage promotes caspase-1 activation and leads to increased expression of IL-1β/IL-18, inducing and exacerbating aseptic inflammatory response (Ma et al., 2014; Feng et al., 2015; Yang et al., 2015; Yuan et al., 2015).

Feng et al. (2015) used the type VII collagenase in SD rats induced haemorrhage model. They found that the microglial cells around the hematoma ion channel P2X7 receptors (P2X7R), NLRP3/ASC/caspase 1, IL-1β/IL-18 higher expression. When using small interfering RNA (siRNA) to silence the P2X7R gene, the expression level is reduced, and it can significantly reduce brain edema and improve neurological deficits. ONOO^−^ as a downstream signaling molecule of P2X7R may play a key role in inducing NLRP3 inflammasome activation. The possible mechanism is as follows: 1) mitochondrial DNA is released, and NLRP3 inflammasome is activated after mitochondrial injury by ONOO-oxidation; 2) NLRP3 nitration led to the separation of TXNIP protein and thiodoxin protein, and TXNIP combined with NLRP3 activated its inflammasome pathway 3) ONOO^−^ may activate NLRP3 (Yuan et al., 2015) by enhancing potassium outflow. Ma et al. (2014) used an intracerebral haemorrhage model induced by injection of autogenous arterial blood in mice. They found that the increase of NLRP3/caspase-1 and IL-1β was accompanied by the formation of mitochondrial permeability transition hole and the increase of mitochondrial reactive oxygen species.

Yang et al. (2015) found in C57BL/6 mouse cerebral haemorrhage model that microRNA-223 (mir-223) could regulate NLRP3 expression and affect inflammatory response. Yuan et al. (2015) found that *in vitro*, the use of recombinant adenovirus-encoded NLRP3 interfering RNA (NLRP3 RNAi) inhibited the inflammatory response induced by microglia (decreased secretion of IL-1β, IL-6, TNF-α), and increased neuronal activity and decreased neuronal apoptosis.

Recent studies (Yao et al., 2016) have shown that NLRP3 signaling pathway expression is gradually up-regulated in tissues around hematoma 1–5 days after ICH. NLRP3 is involved in complement induced neuroinflammatory response, which ultimately leads to neurological dysfunction. For example, Ma et al. (2014) injected autologous blood from mice into ICH model. They found that ROS was the main factor activating NLRP3 inflammasomes. Ye et al. (2017b) also demonstrated that the ROS/TXNIP pathway is involved in thromase-induced NLRP3 inflammasome activation and apoptosis in BV2 microglia exposed to thrombin. Yang et al. (2015) confirmed by Target Scan and dual-luciferase reporting and analysis system that microRNA-223 can directly regulate NLRP3 expression, inhibit the inflammatory response, reduce brain oedema and improve neurological function and behavioral scores of ICH model mice by directly targeting its 3′ non-translation region (3′-UTR). Another study found that the membrane of purine receptor 7 (P2X7R) upstream of NLRP3 activation, can induce NLRP3 inflammasome dependence of pro-inflammatory cytokines, such as IL-1β/IL-18). This relates the inflammation and the nerve damage in a rat ICH model. P2X7R injection through specific inhibitors of brilliant blue G (BBG) inhibited P2X7R/NLRP3 inflammatory reaction after ICH, for example, can effectively reduce brain oedema, and improve the function of ICH after (Feng et al., 2015). In addition, Yuan et al. (2015) used RNAi gene knockdown technology to reduce NLRP3 transcription and inhibit the inflammatory response caused by erythrocyte lysis, thereby reducing microglia-mediated neuron damage and improving the prognosis of ICH patients.

#### NLRP3 Inflammasome and Subarachnoid Hemorrhage

SAH is another type of stroke with a complex pathological mechanism, which can trigger inflammation, apoptosis, oxidative stress and other pathological processes, leading to early brain injury (EBI) and delayed brain injury (DBI) (Macdonald, 2014). Shao et al. (2016) found that hydrogen-rich saline therapy can reduce the inflammatory response and cell damage in EBI by inhibiting the activation of NF-κB and the formation of NLRP3 inflammatory vesicles. Other studies have shown that minocycline (Li et al., 2015) and melatonin (Dong et al., 2016) can reduce ROS production and reduce the activation of NLRP3 inflammatory cells after SAH, playing a neuroprotective role against EBI. In addition, Cao et al. (2017) found that melatonin therapy in the SAH model of rats promoted mitochondrial autophagy, inhibited NLRP3 inflammasome activation, reduced pro-inflammatory cytokine levels, and reduced EBI.

#### NLRP3 Inflammasome and Hemorrhagic Transformation After Stroke

Hemorrhagic transformation (HT) after ischemic stroke is a common complication in patients with ischemic stroke. It is usually exacerbated after thrombolytic therapy (Jickling et al., 2014). Multiple molecular mechanisms, such as an increase in matrix metalloproteinases, oxidative stress, and increased inflammatory response, damage the blood-brain barrier and lead to HT (Bian et al., 2015; Imai et al., 2016; Kuroki et al., 2016) after ischemic stroke. Imai et al. (2016) demonstrated that NLRP3 inflammasome plays a key role in HT’s pathologic process after middle cerebral artery ischemia in hyperglycemic rats. Hyperbaric oxygen preconditioning prevents blood-brain barrier destruction and attenuates HT with hyperglycemic exacerbation by blocking the ROS/TXNIP/NLRP3 pathway.

## Conclusion

NLRP3 inflammasome is a multi-molecular complex in the cytoplasm that controls the processing of caspase-1 and the maturation of pro-inflammatory cytokines (such as IL-1b and IL-18), as the most studied inflammasome, it is closely related to a variety of inflammatory diseases and plays an important role in stimulating and regulating innate immunity and inflammatory response. When the NLRP3 inflammasome is activated, it can produce biologically active cytokines IL-1β and IL-18, which mediate a series of pathophysiological processes.

Clinical studies (Alcocer-Gómez et al., 2014) found that the components of NLRP3 inflammasome and the effect product IL-1β of peripheral blood mononuclear cells in patients with depression were significantly higher than those of normal people, and these abnormal indicators will gradually become normal after receiving antidepressant treatment, the results of this study clearly show the relationship between depression and NLRP3. Significant changes in NLRP3 have been found in the central and peripheral studies of animal depression models (Su et al., 2017), and it is speculated that NLRP3 inflammasome may be an important target for the treatment of depression. Therefore, NLRP3 has very important clinical value in the screening, early recognition and treatment of depression. In addition, because there are many studies on the role of IL-1β in depression, there are relatively few studies on IL-18, another effect product of the NLRP3 inflammasome, exactly what role IL-18 can play in the pathogenesis of depression and whether there is an interaction between IL-1β and IL-18 is still inconclusive. Therefore, related research on IL-18 is expected to become another important direction of depression research.

Researchers first proposed in 2008 that after incubating mouse primary MG with Aβ, NLRP3 inflammasome was activated, and the phagocytosis of fibrotic Aβ by MG can cause the swelling and rupture of lysosomes and release cathepsin B, and promote the mature release of IL-1β, TNF-α and other inflammatory factors and the production of chemokines; the initiation of NLRP3 inflammasome in MG also depends on the activation of IL-1β and caspase-1 (Halle et al., 2008; Salminen et al., 2008). Since then, Heneka et al. (2013) further confirmed that NLRP3 inflammasome activation can promote Aβ deposition and AD pathological process in APP/PS1 double transgenic mice, the NLRP3 gene knockout mice can significantly alleviate the spatial memory impairment and promote the elimination of Aβ. That is to say, in the process of AD, Aβ can activate MG to produce NLRP3 inflammasomes, thereby mediating the polarization of MG to the M1 phenotype, which manifests as neuroinflammation such as neuron loss, Aβ clearance disorder and memory loss; when the NLRP3 or caspase-1 in MG is specifically knocked out, MG is transformed into the M2 phenotype, which is manifested as the neuroprotective effect of increased Aβ clearance, spatial memory and tissue remodeling (Tobias et al., 2013). In summary, NLRP3 inflammasome plays an important role in AD-related neuroinflammatory response.

On the one hand, the activation of NLRP3 inflammasomes produces high levels of inflammatory cytokines, recruits other immune cells, and induces an inflammatory response to eliminate the risk-related molecular pattern protein (DAMP) after hemorrhagic stroke; On the other hand, the excessive activation of NLRP3 inflammasomes can lead to persistent inflammation and brain damage after intracranial hemorrhage. Therefore, studying the role of NLRP3 inflammasome in related mechanisms after intracranial hemorrhage will provide a new strategy for its treatment. It is worth noting that many important issues regarding the pathogenesis of NLRP3 inflammasomes in intracranial hemorrhage need to be further studied and elucidated, such as the activation mechanism and effects of NLRP3 inflammasomes in the late stage of cerebral hemorrhage. In addition, the existing evidence shows that NLRP3 expression is increased in microglia and NLRP3 inflammasome is activated, which plays an important role in the pathological process of cerebral hemorrhage. However, whether the NLRP3 inflammasome has the same effects on neurons and astrocytes still needs further research.

The inflammatory response after ischemic stroke is a complex process involving the activation of innate immune response and the infiltration of white blood cells in the circulation. NLRP3 also plays an important role in this process. Targeted intervention on NLRP3’s activation mode, action mode and itself is the current research hotspot for the treatment of inflammatory response after ischemic stroke.

In summary, under different pathological processes and different activation stimuli, the activation and regulation of NLRP3 inflammasomes are diverse, and NLRP3 inflammasomes play a positive or negative role in the disease. For the treatment of NLRP3-mediated diseases, neutralizing antibodies against IL-1β and IL-18 are usually used, the development of drugs that inhibit pro-inflammatory factors such as IL-1β targeted inhibitors has also become a research hotspot in the adjuvant treatment of neurological diseases, but the effect is not ideal. This suggests that the treatment of diseases that target inflammasomes should not only target their downstream products, but should consider the upstream links of inflammasome activation (Shao et al., 2015). Therefore, the discussion of the NLRP3 inflammasome regulatory network and mechanism of action will not only help to understand the physiological and pathological process of inflammation, but also help to provide experimental evidence for targeted treatment of related diseases.
